# Methylation Profiling of Specific Genes in Ependymomas

**DOI:** 10.5146/tjpath.2021.01565

**Published:** 2022-09-15

**Authors:** Naz Kanıt, Pelin Yalçın, Serhat Erbayraktar, Erdener Ozer

**Affiliations:** Department of Molecular Medicine, Dokuz Eylul University Institute of Health Sciences, Izmir, Turkey; Department of Medical Biology, Dokuz Eylul University Institute of Health Sciences, Izmir, Turkey; Department of Neurosurgery, Dokuz Eylul University School of Medicine, Izmir, Turkey; Department of Pathology, Dokuz Eylul University School of Medicine, Izmir, Turkey

**Keywords:** DNA methylation, Ependymoma, Epigenetics, Pyrosequencing

## Abstract

***Objective:*** Ependymomas are neuroepithelial tumors of the central nervous system with heterogeneous biology and clinical course. The aim of the present study is to investigate the relationship between the methylation status and clinicopathological parameters in ependymomas.

***Material and Method:*** DNA methylation status of *CDKN2A, RASSF1A, KLF4* and *ZIC2* genes were quantitatively analyzed with pyrosequencing in 44 ependymoma tumor tissues. The relationship of methylation profiles with tumor subtype, histological grade and patient age was statistically analyzed.

***Results:*** DNA methylation analyses for *CDKN2A* revealed no difference in methylation levels. Of the 31 included samples for optimal *ZIC2* methylation analysis, 10 were hypermethylated (32.3%) and this change was significantly found in the adult spinal ependymomas (p=0.01). *KLF4* hypermethylation was observed in 6 of the overall included 35 samples (17.1%); however, there was no statistically significant relation of the methylation status with tumor subtype, histological grade or age group. *RASSF1A* hypermethylation was observed in overall 40 included samples with varying methylation levels. Higher levels of hypermethylation were significantly related to the grade 3 histology (p=0.01) and spinal ependymomas (p=0.006). The pediatric cases with grade 3 ependymomas and ependymomas of adulthood showed significantly increased *RASSF1A* hypermethylation levels (p<0.001 and p=0.001, respectively).

***Conclusion:*** DNA methylation changes are likely to have biological importance in ependymomas. Both *ZIC2* and *RASSF1A* methylation status may be useful parameters in the subclassification of these tumors.

## INTRODUCTION

Ependymomas are neuroepithelial tumors of the central nervous system (CNS) located both in the brain and spinal cord (1). They are often seen at pediatric ages and demonstrate low survival rates (2). The grading system basically depends on the histology of the tumor; classical ependymomas are considered as grade 2, whereas ependymomas with anaplastic histology are classified as grade 3 tumors. However. the term “anaplastic ependymoma” is no longer used in the 2021 WHO classification of CNS tumors. The current classification divides ependymomas into molecular groups with the supratentorial, posterior fossa, and spinal anatomic location. When molecular analysis fails or is unavailable, the NOS suffix should be used (3).

Ependymoma is a very genetically heterogeneous cancer, and even though a number of whole genome sequencing and global DNA methylation profiling studies have focused on the prognosis and predicting positive outcome for the patients, an epigenetic changes-based risk classification has not yet been established (4,5). In a recent landmark study by Pajtler et al. (5) using a global DNA methylation analysis for 500 cases, three major groups of supratentorial, infratentorial and spinal were defined according to the location of the tumor where each group has three subgroups. However, they did not determine any specific marker for each subgroup. Consequently, all ependymomas are managed with standard treatment, which causes serious side effects for many of the patients (3). This situation underlines the need for determination of molecular parameters, which can be used as prognostic markers in the precision medicine and potential druggable targets.

Studies on the molecular oncogenesis of ependymomas are basically based on the changes of DNA methylation patterns. In the present study, we aimed to investigate the methylation status of the *RASSF1A*, *CDKN2A*, *KLF4* and *ZIC2* genes and to address the relationship of the methylation profiling of the selected genes with tumor subtype, histological grade or age group, as well as to highlight their significance in precision medicine.

## MATERIALS and METHODS

The present study enrolled archival formalin-fixed paraffin-embedded ependymoma tumor tissues of 44 patients treated in our institution from 2000 to 2020 after obtaining approval in accordance with the regulations of the local institutional ethical committee. The clinical and demographic data of the corresponding patients including tumor site, age and gender were obtained from electronic records. The inclusion criteria were sufficient formalin-fixed paraffin-embedded (FFPE) archival tumor tissue and available clinical data including age and tumor localization. Original hematoxylin and eosin (H&E) stained histological slides of the tumors were reviewed by an expert pathologist (E.O.) to confirm the histological diagnosis of ependymoma and select the optimal tissue block for further molecular analyses. Ependymomas were graded and classified according to the 2021 WHO classification.

DNA methylation status of the *CDKN2A*, *RASSF1A, KLF4 *and *ZIC2* genes were quantitatively analyzed with pyrosequencing. Each step for DNA isolation and bisulfite conversion was performed as instructed in the manufacturer’s protocol. Ten 5μ sections from each tumor block were prepared to isolate total genomic DNA using the QIAamp DNA FFPE Tissue Kit (Qiagen, Germany). The bisulfite conversion of the isolated genomic DNA of 20 mL volume for each case was carried out using the EpiTect Bisulfite kit (Qiagen, Germany) to measure the DNA methylation. The bisulfite treated and commercially available control DNAs (EpiTect PCR Control DNA Set, Qiagen, Germany) were used as templates for polymerase chain reaction (PCR) (PyroMark PCR Kit, Qiagen, Germany). The target sequences were amplified using specific biotinylated primers (Cat. no. PM00139993, PM00013293, PM00039550 and PM00054131; PyroMark CpG Assay, Qiagen, Germany). PCR products were then used to generate single stranded DNAs of the amplified sections and captured with streptavidin-coated sepharose beads in corresponding buffers of PyroMark Q24 Advanced CpG Reagents (Qiagen, Germany). The remainders were washed in the PyroMark Q24 workstation (Qiagen, Germany). The single stranded DNAs were incubated with the pyrosequencing primers and sequenced in the PyroMark Q24 platform (Qiagen, Germany). The methylation levels were quantified as T to C percentages and the average values of each CpG site methylation ratios were calculated. Average values below 8% were considered as unmethylated (6,7). The methylation levels of *RASSF1A* were grouped into four quartiles as suggested in a previous study, where methylation levels less than 45.3% were named as Q1, those between 45.3% and 55.08% as Q2, those between 55.08% and 61.42% as Q3, and those higher than 61.42% as Q4 (8).

Statistical analysis was performed with the IBM SPSS Statistics v.22 software in order to display the relation between the methylation status and clinicopathological data using the chi-squared test. The probability level of 0.05 or less was chosen to represent statistical significance. All *p*-values were two-sided and denoted by *p*. Fisher’s exact test was used to calculate *p* values, as the cell frequencies were too small for the standard chi-squared test to be accurate.

## RESULTS

Overall 44 ependymoma tumor tissues were analyzed in the present study. They were non-recurrent tumors and totally excised. Twenty patients were female (45.5%) and 24 were male (54.5%), ranging from 1 to 74 years old (mean: 25.5 ±20.9), and including 20 pediatric (45.5%) and 24 adult (54.5%) cases. Based on tumor classification, there were three groups: supratentorial ependymoma, NOS (n=8, 18.2%), posterior fossa ependymoma, NOS (n=19, 43.2%), and spinal ependymoma, NOS (n=17, 38.6%). Grade 2 histology was observed in 56.8% of the cases (n=25) and the remaining cases showed grade 3 histology (n=19, 43.2%).

**Figure 1:**
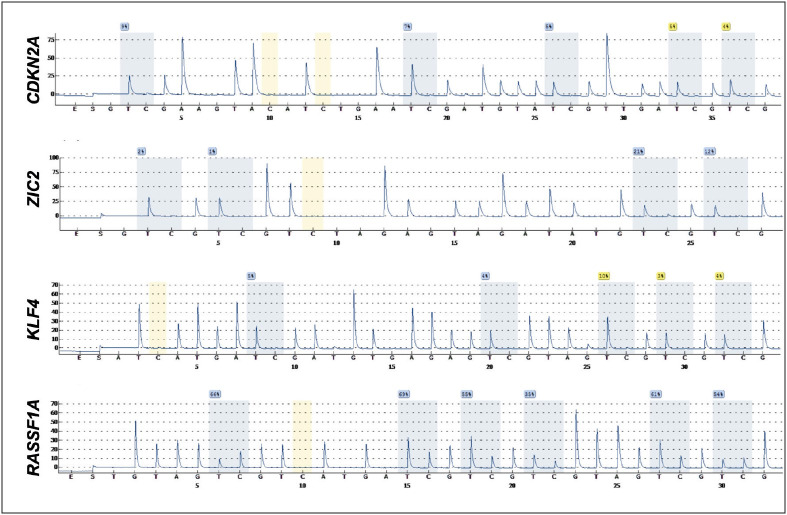
Pyrograms for the target sequences of the CDKN2A, ZIC2, KLF4 and RASSF1A genes.

The pyrosequencing method was utilized to analyze DNA methylation levels of the four selected genes (Figure 1) and the samples with weak signals were excluded. For the *CDKN2A* methylation analysis, 18 samples were included in the study, and hypermethylation was not detected in any case. Of the 31 included samples for optimal *ZIC2* methylation analysis, 10 were hypermethylated (32.3%) and this change was significantly found in the adult spinal ependymomas, NOS (*p*=0.01). *KLF4 *hypermethylation was observed in six of the overall included thirty-five samples (17.1%), however, there was no statistically significant relation of the methylation status with tumor subtype, grade or age group. *RASSF1A* methylation was observed in overall 40 included samples with varying methylation levels; therefore, four quartiles representing the mean methylation values were determined, and demonstrated in Table 1. Higher levels of hypermethylation were significantly related to the grade 3 histology (*p*=0.01) and spinal ependymomas, NOS (*p*=0.006). The pediatric cases with grade 3 ependymomas and adults with spinal ependymoma, NOS showed significantly increased hypermethylation levels (*p*<0.001 and *p*=0.001, respectively).

**Table 1:** *RASSF1A* methylation levels in quartiles and their frequencies related to the histological grade, tumor subtype, and age group.

**Quartiles**			**Q1**	**Q2**	**Q3**	**Q4**
**Histological grade**		Grade 2 (n=24)	9	7	6	2
		Grade 3 (n=16) *	1	3	4	8
**Tumor subtype**		Supratentorial (n=8)	2	2	3	1
		Posterior fossa (n=17)	4	3	3	7
		Spinal (n=15)	4	5	4	2
**Age Group**	Pediatric (n=18)	Grade 2 (n=7)	4	3	0	0
		Grade 3 (n=11) *	0	0	4	7
	Adult (n=22)	Supratentorial (n=9)	5	4	0	0
		Posterior fossa (n=2)	1	1	0	0
		Spinal (n=11) *	0	2	6	3

* Indicates statistical significance of higher hypermethylation levels.

## DISCUSSION

The methylation of *RASSF1A*, *HIC1 *and *CDKN2A *are the most common epigenetic changes demonstrated in ependymomas (1,9,10). Other genes such as *MGMT,* *BLU, GSTP1, DAPK, FHIT, MGMT, MCJ, RARB, TIMP3, THBS1, TP73, CASP8, TFRSF10C *and *TFRSF10D *have also been shown with altered DNA methylation patterns, yet these changes have not been correlated to clinical setting (11,12). In the present study, we analyzed the gene-specific methylation profiles of the *CDKN2A*, *RASSF1A, KLF4 *and *ZIC2 *genes in the ependymoma tumor tissues. The *RASSF1A *and *CDKN2A *genes were selected for analysis in this study since methylations of these genes were previously reported in ependymomas (1,10). In a previous study, lower expression levels of *ZIC2 *were reported in spinal ependymomas in comparison to intracranial ependymomas (13). We therefore investigated the relationship of this genetic change with DNA methylation. On the other hand, we selected the *KLF4* gene because it is widely expressed in neural stem cells, some of which are shown in the ependymomas, and plays a significant role in their self-renewal, also involved in reprogramming of somatic cells to pluripotency (14). In the present study, DNA methylation analyses were performed using bisulfite-based pyrosequencing, which is the gold standard method (15). We found specific DNA methylation patterns exhibiting a relationship with age groups, tumor subtype and grade 3 histology.

The genetic and epigenetic basis of the oncogenesis of ependymomas has been under scrutiny for the last decade. The molecular markers that are associated with the subgroups of ependymomas or related to the patient age are not yet well known. To the best of our knowledge, intracranial and spinal ependymomas are likely to have different genetic and epigenetic signatures. A meta-analysis by Lee et al. (16) has revealed that *NF2* mutations were associated with spinal ependymomas whereas *HIC1* methylation and *EPB41L3* deletions were common in intracranial ependymomas.

The 2021 WHO classification of CNS tumors divides ependymomas into molecular groups with the supratentorial, posterior fossa, and spinal anatomic location. It also lists genetically defined subtypes of supratentorial, posterior fossa and spinal ependymomas. When molecular analysis fails or is unavailable, the NOS suffix should be used, as we did in our study. In addition, according to the current classification, a pathologist can still grade the ependymomas as either CNS WHO grade 2 or grade 3; however the term “anaplastic ependymoma” is no longer listed (3).

In the present study, we analyzed the DNA methylation levels of *CDKN2A*, which is a tumor suppressor gene, and widely modified in a range of cancers (17–20). High-grade gliomas display decreased *CDKN2A* expression levels (21), whereas *CDKN2A* mutations were observed in the recurrent meningiomas (22). Although the DNA methylation analysis revealed unmethylated status for* CDKN2A* in all ependymoma cases, there are contrasting results in the literature. In a study with 123 cases, Rousseou et al. (10) revealed that 21% of all ependymomas showed *CDKN2A* promoter methylations, whereas no methylation was shown in another study similar to our study (23). We therefore think that further research is needed in order to address the importance of promoter methylations of *CDKN2A* in ependymomas.

*ZIC2 *is another gene that has been previously shown to regulate the development of neural tissues and its mutation and downregulation have been linked to neurodevelopment (24). Alterations of this gene have been found to have a crucial function in pediatric medulloblastoma pathogenesis (25), as well as in a broad spectrum of cancers (26–28). In this study, we showed that the *ZIC2 *gene is hypermethylated significantly in adult spinal ependymomas. Kim et al. (13) have reported that *ZIC2 *expression was downregulated in the spinal ependymomas; however, the mechanism underlying its downregulation, which is likely to be due to DNA methylations, was not specifically analyzed. Although the number of hypermethylated cases in our study is limited, the association between *ZIC2 *methylations and spinal ependymomas in adulthood may be a potential mechanism of oncogenesis.

*KLF4* encodes a tumor suppressor and its methylation can lead to silencing of the gene, which has been reported in variety of cancers such as pancreatic cancer, leukemia, colorectal cancer, and medulloblastoma (29–32). We analyzed herein the methylation levels of *KLF4 *and 17.1% of our ependymoma tissues were hypermethylated. However, we did not observe a significant relationship of *KLF4 *hypermethylation with tumor location, patient age or histology of the tumor. We think that the methylation status of *KLF4 *may play a role in the oncogenesis of ependymomas but further studies with wider series are needed to address whether *KLF4 *hypermethylation can be an epigenetic signature.

Hypermethylation of the *RASSF1A* promoter is one of the most common molecular changes in ependymomas (1). In this study, varying hypermethylation levels of this gene were analyzed in terms of quartiles in order to define a cut-off in which the methylation has a significant relationship with the prognosis. Higher *RASSF1A* methylations were observed in pediatric grade 3 ependymomas, and it may therefore be related to a worse prognosis. Adult spinal ependymomas showed significantly increased *RASSF1A* methylation levels. These results may explain why ependymomas are genetically subclassified depending on anatomic localization. Although epigenetic subclassification of ependymomas is not yet fully established, we underline the potential importance of *RASSF1A* promoter methylations as a molecular marker for classification.

Current cancer research is mainly focused on discovering biomarkers, which can potentially be targets for drug selection or to stratify the patients according to the risk categories. Although there are some important limitations in our study such as the limited number of positive cases and insufficient prognostic follow-up data, we think DNA methylation changes could have a biological significance in ependymomas. Both *ZIC2 *and *RASSF1A *methylation status may be useful parameters in the subclassification of ependymomas. In addition, *RASSF1A *hypermethylation level may play a biological role in pediatric ependymomas, also a candidate druggable target changes in grade 3 histology. Because current understanding of ependymomas is limited and there is much need to discover the epigenetic setting of the disease, we think that further studies with wider series will provide sufficient knowledge for the epigenetic changes-based subclassification of ependymomas.

## Conflict of Interest

None of the authors have any conflict of interest
